# Impact of Aurora Kinase A Polymorphism and Epithelial Growth Factor Receptor Mutations on the Clinicopathological Characteristics of Lung Adenocarcinoma

**DOI:** 10.3390/ijerph17197350

**Published:** 2020-10-08

**Authors:** Po-Jen Yang, Ming-Ju Hsieh, Chun-I Lee, Chi-Hua Yen, Hsiang-Ling Wang, Whei-Ling Chiang, Tu-Chen Liu, Thomas Chang-Yao Tsao, Chia-Yi Lee, Shun-Fa Yang

**Affiliations:** 1 School of Medicine, Chung Shan Medical University, Taichung 402, Taiwan; cshy1030@csh.org.tw (P.-J.Y.); cshy352@csh.org.tw (C.-H.Y.); his885889@gmail.com (T.C.-Y.T.); 2 Department of Family and Community Medicine, Chung Shan Medical University Hospital, Taichung 402, Taiwan; 3 Institute of Medicine, Chung Shan Medical University, Taichung 402, Taiwan; 170780@cch.org.tw (M.-J.H.); adoctor0402@gmail.com (C.-I.L.); 4 Cancer Research Center, Changhua Christian Hospital, Changhua 500, Taiwan; 5 Graduate Institute of Biomedical Sciences, China Medical University, Taichung 404, Taiwan; 6 Division of Infertility, Lee Women’s Hospital, Taichung 406, Taiwan; 7 Department of Beauty Science, National Taichung University of Science and Technology, Taichung 404, Taiwan; charmaine790520@yahoo.com.tw; 8 School of Medical Laboratory and Biotechnology, Chung Shan Medical University, Taichung 402, Taiwan; wlchiang@csmu.edu.tw; 9 Department of Chest Medicine, Cheng-Ching General Hospital, Taichung 407, Taiwan; liou.dj@msa.hinet.net; 10 Division of Chest, Department of Internal Medicine, Chung Shan Medical University Hospital, Taichung 402, Taiwan; 11 Department of Ophthalmology, Show Chwan Memorial Hospital, Changhua 500, Taiwan; ao6u.3msn@hotmail.com; 12 Department of Medical Research, Chung Shan Medical University Hospital, Taichung 402, Taiwan

**Keywords:** AURKA, epidermal growth factor receptor, single-nucleotide polymorphism, lung adenocarcinoma

## Abstract

Lung adenocarcinoma (LADC) is the most common subtype of lung cancer worldwide and the epidermal growth factor receptor (EGFR) has a great influence on its clinical course, mainly due to the influence of different phenotypes. The Aurora kinase A (AURKA) would influence the progression of several solid malignancies. However, whether the interaction between EGFR phenotypes and AURKA would influence the clinical characteristics of LADC remains unknown. Herein, this study aimed to explore the effects of single-nucleotide polymorphisms (SNPs) of AURKA and EGFR phenotypes on the clinicopathological characteristics of LADC. Four loci of AURKA SNPs (*rs1047972, rs2273535, rs6024836*, and *rs2064863*) were genotyped using TaqMan allelic discrimination in 105 wild-type EGFR individuals and 167 LADC patients with EGFR mutations. After the statistical analysis, patients with LADC who had CT heterozygotes of AURKA *rs1047972* had a lower risk of EGFR mutations than patients with wild-type homozygotes. Moreover, female and nonsmoking patients who carried the CT genotype of AURKA *rs1047972* had a lower risk of EGFR mutation (*p* = 0.008 and *p* = 0.004, respectively). Moreover, in patients with EGFR mutations, AURKA SNP *rs6024836* G allele (AG + GG) carriers had a lower risk of developing advanced-stage LADC (stage III or IV; odds ratio = 0.423, 95% confidence interval: 0.203–0.879, *p* = 0.019) than patients with AA homozygotes. Our results suggested that AURKA *rs1047972* variants are significantly associated with EGFR mutations among patients with LADC, particularly in female and nonsmoking patients. AURKA variants may contribute to the pathological development of LADC.

## 1. Introduction

Lung adenocarcinoma (LADC) is the most common subtype of lung cancer in both male and female patients [1]. The global incidence rate (per 100,000 individuals) of LADC is 1.4 to 20.7 in males and 0.4 to 12.6 in females [2], and the female predominance of LADC over other lung cancers is noted globally and is the reason for LADC cases outnumbering those of lung squamous cell carcinoma [3]. Conventional therapies for LADC include chemotherapy, radiotherapy, and surgical excision [4]. Recently, the possibility of a therapy targeting the specific tumor proteins has been investigated. For example, methyl-β-cyclodextrin can enhance the effect of doxorubicin on treating breast and liver cancers via influencing the p53 and Fas receptor ligand complex [5], and c-Fos expression may relate to the development of head and neck squamous cell carcinoma [6]. Moreover, tumor heterogeneity may also be a target of treatment in breast cancer [7]. Specifically, target therapies that focus on specific phenotypes of LADC have been developed, and they target the mutation sites of tumor cells that include receptor tyrosine kinases, angiogenesis pathways, and the apoptosis process [4].

The epidermal growth factor receptor (EGFR) is a crucial protein in LADC treatment because it enables EGFR phenotypes and mutations to become prognostic predictors and treatment targets against LADC [8,9,10]. Common EGFR mutations include *L858R* expression and *Exon 19* in-frame deletion, which can alter the prognosis and overall survival of individuals with LADC [8,11]. In addition to mutations, interactions between EGFR mutations and other genetic variations influence clinicopathological characteristics and LADC prognoses [12]. For example, endothelial nitric oxide synthase polymorphisms and EGFR mutations accelerate lymph node invasion in LADC [13]. Furthermore, certain single-nucleotide polymorphisms (SNPs) of carbonic anhydrase 9 are associated with lower tumor stages and less lymph node involvement in LADC with wild-type EGFR [14]. Accordingly, the SNPs of other proteins may reveal a relationship with EGFR phenotypes that can alter the clinicopathological characteristics of LADC.

Aurora kinase A (AURKA) is a protein that regulates centromere and cell mitosis, affecting the progression of several neoplasms [15,16,17,18]. AURKA SNPs reduce the risk of large tumors in patients with hepatocellular carcinoma [18] and are protective factors against urothelial cell carcinoma [17]. Nevertheless, little research has been conducted on the possible interaction between AURKA SNPs and EGFR mutations. Moreover, because AURKA can enhance the resistance of lung cancer to third-generation EGFR inhibitors [19], a dual effect of AURKA SNPs and EGFR mutations on the clinicopathological characteristics of LADC may occur, thus requiring evaluation.

The current study surveyed the association between AURKA SNPs and susceptibility to EGFR mutations in patients with LADC. Furthermore, the synthetic effect of AURKA SNPs and EGFR mutations on the clinicopathological characteristics of LADC was investigated.

## 2. Materials and Methods

### 2.1. Study Subjects and Ethics Statement

The study was conducted in Cheng Ching Hospital and Chung Shan Medical University Hospital. Patients with LADC in a follow-up period longer than 1 year from either hospital were enrolled in the study group. A total of 272 patients with LADC were included. Medical records were obtained with demographic data that included the age, sex, and smoking habits of participants. Furthermore, tumor, node, and metastasis (TNM) status and tumor stage were defined according to the method described in the American Joint Committee on Cancer manual. The current study was approved by the Institutional Review Boards of Cheng Ching Hospital. Written informed consent was obtained from all participants in the current study.

### 2.2. Genomic DNA Extraction and EGFR Sequencing

The DNA extraction and sequencing of EGFR was performed according to previous experience [13]. Tumor tissue from frozen specimens was used to extract DNA by using the QIAamp DNA Kit (Qiagen, Valencia, CA, USA) according to the manufacturer’s guidelines. After the DNA genome was obtained, the categories of EGFR, including wild-type and mutated variants, were classified using a DNA-sequencing reaction (Applied Biosystems, Foster City, CA, USA).

### 2.3. Genotyping of AURKA SNPs from Real-Time Polymerase Chain Reactions

Four AURKA SNPs, namely *rs1047972* (C/T), *rs2273535* (T/A), *rs6024836* (A/G), and *rs2064863* (T/G), were selected due to their considerable effects on other malignancies [16,17,18]. Regarding genotyping, DNA was first extracted from the leukocytes of venous blood samples from each participant using the QIAamp DNA kits (Qiagen, Valencia, Valencia, CA, USA) according to the manufacturer’s instructions. Subsequently, the allelic discrimination of the four AURKA SNPs was surveyed using the ABI StepOne Real-Time polymerase chain reaction (PCR) system (Applied Biosystems, Foster City, CA, USA). The findings of the real-time PCR were then analyzed using a Safety Data Sheet v3.0 (Applied Biosystems, Foster City, CA, USA) through the TaqMan assay technique to enhance PCR integrity.

### 2.4. Statistical Analyses

SAS v9.4 (SAS Institute Inc., Cary, NC, USA) was used for statistical analyses in the current study. The Mann–Whitney U test and Fisher’s exact test were used to compare differences in the demographic data, tumor stage, and tumor cell differentiation between wild-type and mutated EGFRs. Multiple logistic regression was used to obtain adjusted odds ratios (AORs) with 95% confidence intervals (CIs) for different AURKA SNP distributions between wild-type and mutated EGFR populations after adjusting for age, sex, and tobacco use. Multiple logistic regression was used to investigate the correlation between clinicopathological characteristics and phenotypes of EGFR with different AURKA *rs6024836* SNPs. A *p* value of ≤0.05 indicated a statistically significant difference.

## 3. Results

Of the study population (i.e., 272 patients), 105 had wild-type EGFR and 167 patients had the mutated EGFR phenotype (Table 1). The mean age of the wild-type EGFR group was 65.52 + 13.47 years, which was similar to that of the mutated EGFR group (65.74 + 13.61 years). However, the mutated EGFR group had a significantly higher female ratio (64.1% vs. 41.9%, *p* < 0.001) and more nonsmokers (77.2% vs. 45.7%, *p* < 0.001). Tumor stage and TNM status between the groups were similar, whereas the mutated EGFR group had a significantly higher rate of clear cell differentiation (12.0% vs. 7.6%, *p* = 0.005; Table 1).

The distribution frequencies of AURKA SNPs between the EGFR groups are listed in Table 2. AURKA SNP *rs1047972* CT (AOR: 0.458, 95% CI: 0.243–0.862, *p* = 0.015) and AURKA SNP *rs1047972* CT + TT (AOR: 0.471, 95% CI: 0.251–0.884, *p* = 0.019) had significantly lower distributions in the EGFR mutation group. However, the distribution frequencies of other AURKA SNPs, including *rs2273535*, *rs6024836*, and *rs2064863*, were not significantly different between the EGFR groups (Table 2). In the subgroup analysis stratified by sex, the female population with EGFR mutations exhibited significantly lower distributions of AURKA SNP *rs1047972* CT (AOR: 0.321, 95% CI: 0.139–0.740, *p* = 0.008) and AURKA SNP *rs1047972* CT + TT (AOR: 0.321, 95% CI: 0.139–0.740, *p* = 0.008; Table 3). Regarding the effect of smoking, nonsmokers with EGFR mutations had significantly lower distributions of AURKA SNP *rs1047972* CT (AOR: 0.331, 95% CI: 0.156–0.703, *p* = 0.004) and AURKA SNP *rs1047972* CT + TT (AOR: 0.349, 95% CI: 0.165–0.737, *p* = 0.006; Table 4).

The clinicopathological characteristics of LADC and its association with different EGFR phenotypes and AURKA SNP *rs6024836* are presented in Table 5. In patients with EGFR mutations, AURKA SNP *rs6024836* G allele (AG + GG) carriers had a lower risk of developing an advanced clinical stage of LADC (stage III or IV; odds ratio = 0.423, 95% CI: 0.203–0.879, *p* = 0.019) than patients with AA homozygotes. However, the distribution frequency of AURKA SNP *rs6024836* did not reveal a significant difference between the entire study group and the wild-type EGFR group concerning tumor stage, TNM status, or cell differentiation condition (Table 5).

## 4. Discussion

Our results revealed that patients with LADC who had CT heterozygotes of AURKA *rs1047972* had a lower risk of EGFR mutations than patients with wild-type homozygotes. Moreover, patients with AURKA SNP *rs6024836* AG + GG had a lower risk of advanced-stage LADC in the mutated EGFR group.

Extensive research has been conducted on the relationship between AURKA and the formation of solid malignancies [20,21,22]. AURKA is a serine–threonine kinase essential for centromere maturation, contributing to subsequent mitosis and cytokinesis [20,23,24]. In a previous study, AURKA suppressed degradation transcription factor N-MYC, thereby promoting G1-S progression [25]. However, AURKA is a key factor for T cell activation, which occurs mainly through the Lck signal [26] and involves T cells in the immune reaction against cancer [27]. The genetic polymorphism of AURKA can influence its activity, in which a lower kinase activity of AURKA resulting from the different SNP could lead to genomic instability and neoplasm [28]. As such, the amplification of a specific AURKA genetic variant was observed in colon cancer [29]. For specific malignancy development associated with AURKA polymorphism, AURKA SNP *rs6024836* was correlated with a higher susceptibility to breast cancer after multivariable analysis [30]. In addition, AURKA SNP *rs2273535* significantly increased the risk of gastric tumors, particularly in female patients and nonsmokers [31]. In addition, AURKA polymorphism may influence the prognosis of cancer because the presence of certain AURKA SNPs is associated with longer progression-free survival for advanced urothelial cell carcinoma or other solid tumors [32,33]. Apart from AURKA itself, certain interactions between AURKA and other oncogenic factors affect the progression of cancers [15]. For instance, AURKA contributes to higher tumor grades in estrogen receptor-positive primary breast cancers [34]. Moreover, the coexistence of betel nut chewing and AURKA SNPs is associated with the development of oral squamous cell carcinoma [16]. Regarding the relationship between AURKA and EGFR, a previous study revealed higher AURKA activity in patients with LADC resistance to tyrosine kinase inhibitors that target EGFR mutations [19]. Moreover, the presence of dual inhibitors for AURKA and EGFR kinase has been previously reported [35]. Similar to the SNP of AURKA, the polymorphism of EGFR would alter the function of EGFR: the *L858R* expression of EGFR would decrease cellular growth and migration compared to EGFR wild type [36], while some forms of *Exon 19* in-frame deletion could increase the signal intensity of phosphorylated EGFR [37]. Consequently, AURKA SNPs and EGFR phenotypes may be associated in individuals with LADC and affect the clinical characteristics of LADC, as indicated by the results of the current study.

The distribution frequency of AURKA SNP *rs1047972* was significantly different between patients with LADC with mutated and wild-type EGFR. Little research has analyzed the relationship between the distribution of AURKA SNPs and EGFR mutations, except for certain indirect associations that indicate interaction between AURKA SNPs and EGFR phenotypes [19,35]. Because the EGFR mutations enrolled in the current study (i.e., *L858R* expression and *Exon 19* in-frame deletion) alter tumor progression and deterioration in LADC [8,38,39], a reduced occurrence of AURKA SNP *rs1047972* in the population indicates that AURKA SNP *rs1047972* has similar genetic variations to that of EGFR mutations, which merits further evaluation. However, the other AURKA SNPs did not exhibit such a relationship with mutated EGFR, which may imply a solitary relationship between AURKA SNP *rs1047972* frequency and EGFR mutations rather than with gross AURKA. In the subgroup analysis of AURKA SNP, female patients and nonsmokers in the mutated EGFR group had a significantly lower rate of AURKA SNP *rs1047972* expression. Female patients and nonsmokers are at risk of LADC [40,41,42]. Accordingly, the lower distribution frequency of AURKA SNP *rs1047972* indicates a high-risk group to LADC, from which further research can be conducted.

Regarding the correlation of AURKA and EGFR phenotypes with the clinicopathological characteristics of LADC, the presence of AURKA SNP *rs6024836* and mutated EGFR phenotypes was correlated with an advanced LADC clinical stage at initial presentation. To our knowledge, this was the first experience illustrating the correlation of AURKA SNP variation with the clinical course of LADC. Generally, EGFR mutations such as *L858R* expression and *Exon 19* in-frame deletion indicate favorable treatment outcomes compared with individuals with lung cancer and wild-type EGFR in terms of relapse-free and overall survival [38,39]. Consequently, the increased expression of AURKA SNP *rs6024836* in patients with such EGFR phenotypes indicates the synergetic effects of genetic polymorphism on delayed LADC progression, as well as easier control of LADC in patients with such dual variations that require further validation. Regarding other clinicopathological characteristics of LADC, the distribution frequency of AURKA SNP *rs6024836* was numerically higher in the mutated EGFR group with a lower tumor T status, absence of lymph node involvement, and negative distant metastasis. These results suggested that AURKA SNP *rs6024836* creates its own universal effect in early cancer stages if it coexists with mutated EGFR phenotypes, and a study with more cases is warranted. However, no significant correlation between wild-type EGFR and the distribution frequency of AURKA SNP *rs6024836* for the clinicopathological characteristics of LADC appeared, and the association was nonsignificant when the entire study population with various EGFR phenotypes was evaluated. This phenomenon indicates the relationship of genetic polymorphisms between AURKA and EGFR that may be due to the involvement of cell migration processes from both proteins [24,43].

Regarding demographic and basic characteristics in the wild-type and mutated EGFR mutation populations, the ratios of female patients, nonsmokers, and those with better cell differentiation were observed in patients with LADC and mutated EGFR phenotypes. Female patients are more prone to lung cancer, including LADC [42], and EGFR mutations are predictors of favorable responses to target treatments [11,38]. This result implies that other genetic factors cause women to become vulnerable to LADC. A similar condition occurred regarding smoking habits. Nonsmoking was correlated with the development of LADC, but more nonsmokers had mutated EGFR phenotypes [40]. The clinicopathological characteristics between the two EGFR phenotype groups were largely similar and cell differentiation in both groups was typically moderate. The larger numbers of cases with well-differentiated LADC in the mutated EGFR group may have been related to the favorable response to treatment in this population [11].

There were several limitations in the current study. First, the relatively few case numbers of the current study may diminish the statistical power and let multivariable analysis that includes other co-morbidities become difficult; thus, a study with much larger case numbers should be conducted. On the other hand, the rate of LADC progression cannot be assessed because of the case-control design of the current study; another research work with a cohort design should be made to evaluate the possible relationship between the interaction of EGFR mutations and AURKA SNPs and subsequent progression of LADC. Moreover, the gender ratio and smoking status were not matched in the current study. Nevertheless, since LADC tends to occur more commonly in the female population and nonsmoker in previous experiences [40], this condition might be regarded as a real-world distribution of LADC patients rather than a flaw of the research design.

## 5. Conclusions

In conclusion, the distribution frequency of AURKA SNP *rs1047972* was significantly different in the group with LADC and mutated EGFR, particularly among female patients and nonsmokers. Furthermore, the presence of AURKA SNP *rs6024836* and coexistence with mutated EGFR was correlated with advanced clinical stages of LADC. This evidence indicated that the relationship between AURKA SNP and EGFR genotypes can alter LADC progression and its clinical course. Further large-scale prospective studies of the effects of AURKA SNP and EGFR genotypes on long-term therapeutic outcomes and overall survival from LADC are suggested.

## Figures and Tables

**Table 1:** Clinical characteristics in lung adenocarcinoma patients with epidermal growth factor receptor (EGFR), either wild type or mutation type.

Subject Characteristics	EGFR Wild Type (*n* = 105)	EGFR Mutation Type (*n* = 167)	*p* Value
Age, *n* (%)			
Mean ± SD (years)	65.52 ⩲ 13.47	65.74 ⩲ 13.61	0.897
Gender, *n* (%)			
Male	61 (58.1%)	60 (35.9%)	<0.001
Female	44 (41.9%)	107 (64.1%)	
Cigarette smoking, *n* (%)			
Nonsmoker	48 (45.7%)	129 (77.2%)	<0.001
Ever-smoker	57 (54.3%)	38 (22.8%)	
Stage, *n* (%)			
I + II	24 (22.9%)	47 (28.1%)	0.334
III + IV	81 (77.1%)	120 (71.9%)	
Tumor T status, *n* (%)			
T1 + T2	59 (56.2%)	106 (63.5%)	0.231
T3 + T4	46 (43.8%)	61 (36.5%)	
Lymph node status, *n* (%)			
Negative	27 (25.7%)	52 (31.1%)	0.337
Positive	78 (74.3%)	115 (68.9%)	
Distant Metastasis, *n* (%)			
Negative	52 (49.5%)	80 (47.9%)	0.795
Positive	53 (50.5%)	87 (52.1%)	
Cell differentiation, *n* (%)			
Well	8 (7.6%)	20 (12.0%)	0.005
Moderately	78 (74.3%)	137 (82.0%)	
Poorly	19 (18.1%)	10 (6.0%)	

SD—standard deviation; *n*—number.

**Table 2:** Distribution frequency of Aurora kinase A (AURKA) genotypes of lung adenocarcinoma with different epidermal growth factor receptor phenotypes.

Genotype SNP	EGFR Wild Type (*n* = 105)	EGFR Mutation Type (*n* = 167)	AOR (95% CI)	*p* Value
*rs1047972*				
CC	76 (72.4%)	137 (82.0%)	1.00	
CT	29 (27.6%)	29 (17.4%)	0.458 (0.243–0.862)	0.015
TT	0 (0.0%)	1 (0.6%)	-	-
CT + TT	29 (27.6%)	30 (18.0%)	0.471 (0.251–0.884)	0.019
*rs2273535*				
TT	46 (43.8%)	78 (46.7%)	1.00	
TA	49 (46.7%)	76 (45.5%)	0.782 (0.450–1.360)	0.383
AA	10 (9.5%)	13 (7.8%)	0.688 (0.258–1.837)	0.455
TA + AA	59 (56.2%)	89 (53.3%)	0.766 (0.451–1.302)	0.325
*rs6024836*				
AA	49 (46.7%)	70 (41.9%)	1.00	
AG	41 (39.0%)	74 (44.3%)	1.060 (0.602–1.868)	0.839
GG	15 (14.3%)	23 (13.8%)	0.903 (0.405–2.012)	0.803
AG + GG	56 (53.3%)	97 (58.1%)	1.018 (0.601–1.726)	0.946
*rs2064863*				
TT	72 (68.6%)	113 (67.7%)	1.00	
TG	28 (26.7%)	47 (28.1%)	1.069 (0.590–1.935)	0.826
GG	5 (4.7%)	7 (4.2%)	0.893 (0.246–3.245)	0.863
TG + GG	33 (31.4%)	54 (32.3%)	1.043 (0.593–1.834)	0.883

SNP—single-nucleotide polymorphism; *n*—number; AOR—adjusted odds ratios after controlling for age, gender, and cigarette smoking.

**Table 3:** Distribution frequency of AURKA genotypes of lung adenocarcinoma with different gender and epidermal growth factor receptor phenotypes.

Genotype SNP	Male (*n* = 121)			Female (*n* = 151)		
	EGFR Wild Type (*n* = 61)	EGFR Mutation Type (*n* = 60)	*p* Value	EGFR Wild Type (*n* = 44)	EGFR Mutation Type (*n* = 107)	*p* Value
*rs1047972*						
CC	48 (78.7%)	50 (83.3%)		28 (63.6%)	87 (81.3%)	
CT	13 (21.3%)	9 (15.0%)	0.240	16 (36.4%)	20 (18.7%)	0.008 ^a^
TT	0 (0.0%)	1 (1.7%)	-	0 (0.0%)	0 (0.0%)	-
CT + TT	13 (21.3%)	10 (16.7%)	0.298	16 (36.4%)	20 (18.7%)	0.008 ^b^
*rs2273535*						
TT	30 (49.2%)	30 (50.0%)		16 (36.4%)	48 (44.9%)	
TA	25 (41.0%)	22 (36.7%)	0.608	24 (54.5%)	54 (50.5%)	0.167
AA	6 (9.8%)	8 (13.3%)	0.723	4 (9.1%)	5 (4.7%)	0.145
TA + AA	31 (50.8%)	30 (50.0%)	0.765	28 (63.6%)	59 (55.1%)	0.116
*rs6024836*						
AA	32 (52.5%)	22 (36.7%)		17 (38.6%)	48 (44.9%)	
AG	23 (37.7%)	28 (46.7%)	0.559	18 (40.9%)	46 (43.0%)	0.495
GG	6 (9.8%)	10 (16.6%)	0.173	9 (20.5%)	13 (12.1%)	0.157
AG + GG	29 (47.5%)	38 (63.3%)	0.308	27 (61.4%)	59 (55.1%)	0.278
*rs2064863*						
TT	41 (67.2%)	40 (66.7%)		31 (70.5%)	73 (68.2%)	
TG	18 (29.5%)	15 (25.0%)	0.811	10 (22.7%)	32 (29.9%)	0.687
GG	2 (3.3%)	5 (8.3%)	0.222	3 (6.8%)	2 (1.9%)	0.357
TG + GG	20 (32.8%)	20 (33.3%)	0.816	13 (29.5%)	34 (31.8%)	0.127

SNP—single-nucleotide polymorphism; *n*—number; AOR—adjusted odds ratios after controlling for age and cigarette smoking. CI—confidence intervals; ^a^ AOR (95% CI): 0.321 (0.139–0.740); ^b^ AOR (95% CI): 0.321 (0.139–0.740).

**Table 4:** Distribution frequency of AURKA genotypes of lung adenocarcinoma with different cigarette smoking status and epidermal growth factor receptor phenotype.

Genotype SNP	Non-Smoking (*n* = 177)			Smoking (*n* = 95)		
	EGFR Wild Type (*n* = 48)	EGFR Mutation Type (*n* = 129)	*p* Value	EGFR Wild Type (*n* = 57)	EGFR Mutation Type (*n* = 38)	*p* Value
*rs1047972*						
CC	29 (60.4%)	105 (81.4%)		47 (82.5%)	32 (84.2%)	
CT	19 (39.6%)	23 (17.8%)	0.004 ^a^	10 (17.5%)	6 (15.8%)	0.694
TT	0 (0.0%)	1 (0.8%)	-	0 (0.0%)	0 (0.0%)	-
CT + TT	19 (39.6%)	24 (18.6%)	0.006 ^b^	10 (17.5%)	6 (15.8%)	0.694
*rs2273535*						
TT	17 (35.4%)	60 (46.5%)		29 (50.9%)	18 (47.4%)	
TA	25 (52.1%)	60 (46.5%)	0.167	24 (42.1%)	16 (42.1%)	0.697
AA	6 (12.5%)	9 (7.0%)	0.181	4 (7.0%)	4 (10.5%)	0.512
TA + AA	31 (64.6%)	69 (53.5%)	0.110	28 (49.1%)	20 (52.6%)	0.905
*rs6024836*						
AA	17 (35.4%)	54 (41.9%)		32 (56.1%)	16 (42.1%)	
AG	22 (45.8%)	59 (45.7%)	0.400	19 (33.3%)	15 (39.5%)	0.673
GG	9 (18.8%)	16 (12.4%)	0.171	6 (10.5%)	7 (18.4%)	0.171
AG + GG	31 (64.6%)	75 (58.1%)	0.242	25 (43.9%)	22 (57.9%)	0.351
*rs2064863*						
TT	34 (70.8%)	88 (68.2%)		38 (66.7%)	25 (65.8%)	
TG	11 (22.9%)	37 (28.7%)	0.547	17 (29.8%)	10 (26.3%)	0.732
GG	3 (6.3%)	4 (3.1%)	0.286	2 (3.5%)	3 (7.9%)	0.357
TG + GG	14 (29.2%)	41 (31.8%)	0.820	19 (33.3%)	13 (34.2%)	0.986

SNP—single-nucleotide polymorphism; *n*—number; AOR—adjusted odds ratios after controlling for age and gender. CI—confidence intervals; ^a^ AOR (95% CI): 0.331 (0.156–0.703); ^b^ AOR (95% CI): 0.349 (0.165–0.737).

**Table 5:** Distribution frequency of AURKA rs6024836 genotypes with clinicopathologic characteristics in lung adenocarcinoma patients.

**Variable**	**ALL (*n* = 272)**			
	**AA** **(*n* = 119)**	**AG + GG** **(*n* = 153)**	**OR (95% CI)**	***p* Value**
Stages				
I + II	25 (21.0%)	46 (30.1%)	1.00	*p* = 0.092
III + IV	94 (79.0%)	107 (69.9%)	0.619 (0.353–1.083)	
Tumor T status				
T1 + T2	70 (58.8%)	95 (62.1%)	1.00	*P* = 0.584
T3 + T4	49 (41.2%)	58 (37.9%)	0.872 (0.534–1.423)	
Lymph node status				
Negative	30 (25.2%)	49 (32.0%)	1.00	*p* = 0.219
Positive	89 (74.8%)	104 (68.0%)	0.715 (0.419–1.222)	
Distant metastasis				
Negative	56 (47.1%)	76 (49.7%)	1.00	*p* = 0.669
Positive	63 (52.9%)	77 (50.3%)	0.901 (0.557–1.455)	
Cell differentiation				
Well/Moderately	104 (87.4%)	139 (90.8%)	1.00	*p* = 0.360
Poorly	15 (12.6%)	14 (9.2%)	0.698 (0.323–1.510)	
	**EGFR Wild Type (*n* = 105)**			
	**AA** **(*n* = 49)**	**AG + GG** **(*n* = 56)**	**OR (95% CI)**	***p* Value**
Stages				
I + II	12 (24.5%)	12 (21.4%)	1.00	*p* = 0.709
III + IV	37 (75.5%)	44 (78.6%)	1.189 (0.478–2.960)	
Tumor T status				
T1 + T2	30 (61.2%)	29 (51.8%)	1.00	*p* = 0.331
T3 + T4	19 (38.8%)	27 (48.2%)	1.470 (0.675–3.200)	
Lymph node status				
Negative	12 (24.5%)	15 (26.8%)	1.00	*p* = 0.788
Positive	37 (75.5%)	41 (73.2%)	0.886 (0.368–2.136)	
Distant metastasis				
Negative	28 (57.1%)	24 (42.9%)	1.00	*p* = 0.144
Positive	21 (42.9%)	32 (57.1%)	1.778 (0.819–3.858)	
Cell differentiation				
Well/Moderately	37 (75.5%)	49 (87.5%)	1.00	*p* = 0.111
Poorly	12 (24.5%)	7 (12.5%)	0.440 (0.158–1.228)	
	**EGFR Mutation (*n* = 167)**			
	**AA** **(*n* = 70)**	**AG + GG** **(*n* = 97)**	**OR (95% CI)**	***p* Value**
Stages				
I + II	13 (18.6%)	34 (35.1%)	1.00	*p* = 0.019
III + IV	57 (81.4%)	63 (64.9%)	0.423 (0.203–0.879)	
Tumor T status				
T1 + T2	40 (57.1%)	66 (68.0%)	1.00	*p* = 0.149
T3 + T4	30 (42.9%)	31 (32.0%)	0.626 (0.331–1.185)	
Lymph node status				
Negative	18 (25.7%)	34 (35.1%)	1.00	*p* = 0.199
Positive	52 (74.3%)	63 (64.9%)	0.641 (0.325–1.265)	
Distant metastasis				
Negative	28 (40.0%)	52 (53.6%)	1.00	*p* = 0.082
Positive	42 (60.0%)	45 (46.4%)	0.577 (0.309–1.075)	
Cell differentiation				
Well/Moderately	67 (95.7%)	90 (92.8%)	1.00	*p* = 0.431
Poorly	3 (4.3%)	7 (7.2%)	1.737 (0.433–6.967)	

SNP—single-nucleotide polymorphism; *n*—number.

## List of abbreviations

LADC—lung adenocarcinoma; EGFR—epidermal growth factor receptor; SNP—single-nucleotide polymorphism; AURKA—Aurora kinase A; TNM—Tumor, Node, Metastasis; PCR—polymerase chain reaction; AOR—adjusted odds ratios; CI—confidence intervals; SD—standard deviation; N—number.

## References

[1] Barta J.A., Powell C.A., Wisnivesky J.P. (2019). Global epidemiology of lung cancer. Ann. Glob. Health.

[2] Cheng T.Y., Cramb S.M., Baade P.D., Youlden D.R., Nwogu C., Reid M.E. (2016). The international epidemiology of lung cancer: Latest trends, disparities, and tumor characteristics. J. Thorac. Oncol..

[3] Youlden D.R., Cramb S.M., Baade P.D. (2008). The international epidemiology of lung cancer: Geographical distribution and secular trends. J. Thorac. Oncol..

[4] Molina J.R., Yang P., Cassivi S.D., Schild S.E., Adjei A.A. (2008). Non-small cell lung cancer: Epidemiology, risk factors, treatment, and survivorship. Mayo Clin. Proc..

[5] Mohammad N., Singh S.V., Malvi P., Chaube B., Athavale D., Vanuopadath M., Nair S.S., Nair B., Bhat M.K. (2015). Strategy to enhance efficacy of doxorubicin in solid tumor cells by methyl-β-cyclodextrin: Involvement of p53 and fas receptor ligand complex. Sci. Rep..

[6] Muhammad N., Bhattacharya S., Steele R., Phillips N., Ray R.B. (2017). Involvement of c-fos in the promotion of cancer stem-like cell properties in head and neck squamous cell carcinoma. Clin. Cancer Res..

[7] Kumar B., Chand V., Ram A., Usmani D., Muhammad N. (2020). Oncogenic mutations in tumorigenesis and targeted therapy in breast cancer. Curr. Mol. Biol. Rep..

[8] Siegelin M.D., Borczuk A.C. (2014). Epidermal growth factor receptor mutations in lung adenocarcinoma. Lab. Investig..

[9] Saito M., Shiraishi K., Kunitoh H., Takenoshita S., Yokota J., Kohno T. (2016). Gene aberrations for precision medicine against lung adenocarcinoma. Cancer Sci..

[10] Calvayrac O., Pradines A., Pons E., Mazières J., Guibert N. (2017). Molecular biomarkers for lung adenocarcinoma. Eur. Respir. J..

[11] Inoue T., Matsumura Y., Araki O., Karube Y., Maeda S., Kobayashi S., Chida M. (2018). Epidermal growth factor receptor gene mutation in pleural lavage cytology findings of primary lung adenocarcinoma cases. Ann. Thorac. Cardiovasc. Surg..

[12] Han L., Lee C.K., Pang H., Chan H.T., Lo I.L., Lam S.K., Cheong T.H., Ho J.C. (2017). Genetic predisposition to lung adenocarcinoma among never-smoking chinese with different epidermal growth factor receptor mutation status. Lung Cancer.

[13] Huang C.Y., Hsieh M.J., Wu W.J., Chiang W.L., Liu T.C., Yang S.F., Tsao T.C. (2018). Association of endothelial nitric oxide synthase (enos) polymorphisms with egfr-mutated lung adenocarcinoma in taiwan. J. Cancer.

[14] Yu Y.Y., Chiou H.L., Tsao S.M., Huang C.C., Lin C.Y., Lee C.Y., Tsao T.C., Yang S.F., Huang Y.W. (2020). Association of carbonic anhydrase 9 polymorphism and the epithelial growth factor receptor mutations in lung adenocarcinoma patients. Diagnostics.

[15] Yan M., Wang C., He B., Yang M., Tong M., Long Z., Liu B., Peng F., Xu L., Zhang Y. (2016). Aurora-a kinase: A potent oncogene and target for cancer therapy. Med. Res. Rev..

[16] Chou C.H., Chou Y.E., Chuang C.Y., Yang S.F., Lin C.W. (2017). Combined effect of genetic polymorphisms of aurka and environmental factors on oral cancer development in taiwan. PLoS ONE.

[17] Huang C.H., Chen C.J., Chen P.N., Wang S.S., Chou Y.E., Hung S.C., Yang S.F. (2019). Impacts of aurka genetic polymorphism on urothelial cell carcinoma development. J. Cancer.

[18] Wang B., Hsu C.J., Chou C.H., Lee H.L., Chiang W.L., Su C.M., Tsai H.C., Yang S.F., Tang C.H. (2018). Variations in the aurka gene: Biomarkers for the development and progression of hepatocellular carcinoma. Int. J. Med. Sci..

[19] Shah K.N., Bhatt R., Rotow J., Rohrberg J., Olivas V., Wang V.E., Hemmati G., Martins M.M., Maynard A., Kuhn J. (2019). Aurora kinase a drives the evolution of resistance to third-generation egfr inhibitors in lung cancer. Nat. Med..

[20] Otto T., Sicinski P. (2017). Cell cycle proteins as promising targets in cancer therapy. Nat. Rev. Cancer.

[21] Zhang J., Li B., Yang Q., Zhang P., Wang H. (2015). Prognostic value of aurora kinase a (aurka) expression among solid tumor patients: A systematic review and meta-analysis. Jpn. J. Clin. Oncol..

[22] Li M., Gao K., Chu L., Zheng J., Yang J. (2018). The role of aurora-a in cancer stem cells. Int. J. Biochem. Cell Biol..

[23] Levinson N.M. (2018). The multifaceted allosteric regulation of aurora kinase a. Biochem. J..

[24] Magnaghi-Jaulin L., Eot-Houllier G., Gallaud E., Giet R. (2019). Aurora a protein kinase: To the centrosome and beyond. Biomolecules.

[25] Otto T., Horn S., Brockmann M., Eilers U., Schüttrumpf L., Popov N., Kenney A.M., Schulte J.H., Beijersbergen R., Christiansen H. (2009). Stabilization of n-myc is a critical function of aurora a in human neuroblastoma. Cancer Cell.

[26] Blas-Rus N., Bustos-Morán E., Martín-Cófreces N.B., Sánchez-Madrid F. (2017). Aurora-a shines on t cell activation through the regulation of lck. Bioessays.

[27] Ping Y., Liu C., Zhang Y. (2018). T-cell receptor-engineered t cells for cancer treatment: Current status and future directions. Protein Cell.

[28] Kimura M.T., Mori T., Conroy J., Nowak N.J., Satomi S., Tamai K., Nagase H. (2005). Two functional coding single nucleotide polymorphisms in stk15 (aurora-a) coordinately increase esophageal cancer risk. Cancer Res..

[29] Ewart-Toland A., Briassouli P., de Koning J.P., Mao J.H., Yuan J., Chan F., MacCarthy-Morrogh L., Ponder B.A., Nagase H., Burn J. (2003). Identification of stk6/stk15 as a candidate low-penetrance tumor-susceptibility gene in mouse and human. Nat. Genet..

[30] Ruan Y., Song A.P., Wang H., Xie Y.T., Han J.Y., Sajdik C., Tian X.X., Fang W.G. (2011). Genetic polymorphisms in aurka and brca1 are associated with breast cancer susceptibility in a chinese han population. J. Pathol..

[31] Zhou X., Wang P., Zhao H. (2018). The association between aurka gene rs2273535 polymorphism and gastric cancer risk in a chinese population. Front. Physiol..

[32] Necchi A., Pintarelli G., Raggi D., Giannatempo P., Colombo F. (2017). Association of an aurora kinase a (aurka) gene polymorphism with progression-free survival in patients with advanced urothelial carcinoma treated with the selective aurora kinase a inhibitor alisertib. Invest. New Drugs.

[33] Niu H., Shin H., Gao F., Zhang J., Bahamon B., Danaee H., Melichar B., Schilder R.J., Coleman R.L., Falchook G. (2017). Aurora a functional single nucleotide polymorphism (snp) correlates with clinical outcome in patients with advanced solid tumors treated with alisertib, an investigational aurora a kinase inhibitor. EBioMedicine.

[34] Lykkesfeldt A.E., Iversen B.R., Jensen M.B., Ejlertsen B., Giobbie-Hurder A., Reiter B.E., Kirkegaard T., Rasmussen B.B. (2018). Aurora kinase a as a possible marker for endocrine resistance in early estrogen receptor positive breast cancer. Acta Oncol..

[35] Kurup S., McAllister B., Liskova P., Mistry T., Fanizza A., Stanford D., Slawska J., Keller U., Hoellein A. (2018). Design, synthesis and biological activity of n(4)-phenylsubstituted-7h-pyrrolo[2,3-d]pyrimidin-4-amines as dual inhibitors of aurora kinase a and epidermal growth factor receptor kinase. J. Enzym. Inhib. Med. Chem..

[36] Erdem-Eraslan L., Gao Y., Kloosterhof N.K., Atlasi Y., Demmers J., Sacchetti A., Kros J.M., Sillevis Smitt P., Aerts J., French P.J. (2015). Mutation specific functions of egfr result in a mutation-specific downstream pathway activation. Eur. J. Cancer.

[37] Furuyama K., Harada T., Iwama E., Shiraishi Y., Okamura K., Ijichi K., Fujii A., Ota K., Wang S., Li H. (2013). Sensitivity and kinase activity of epidermal growth factor receptor (egfr) exon 19 and others to egfr-tyrosine kinase inhibitors. Cancer Sci..

[38] Isaka T., Nakayama H., Ito H., Yokose T., Yamada K., Masuda M. (2018). Impact of the epidermal growth factor receptor mutation status on the prognosis of recurrent adenocarcinoma of the lung after curative surgery. BMC Cancer.

[39] Hayasaka K., Shiono S., Matsumura Y., Yanagawa N., Suzuki H., Abe J., Sagawa M., Sakurada A., Katahira M., Takahashi S. (2018). Epidermal growth factor receptor mutation as a risk factor for recurrence in lung adenocarcinoma. Ann. Thorac. Surg..

[40] Pallis A.G., Syrigos K.N. (2013). Lung cancer in never smokers: Disease characteristics and risk factors. Crit. Rev. Oncol. Hematol..

[41] Rivera G.A., Wakelee H. (2016). Lung cancer in never smokers. Adv. Exp. Med. Biol..

[42] De Groot P., Munden R.F. (2012). Lung cancer epidemiology, risk factors, and prevention. Radiol. Clin. N. Am..

[43] Wang Y., Deng W., Zhang Y., Sun S., Zhao S., Chen Y., Zhao X., Liu L., Du J. (2018). Mical2 promotes breast cancer cell migration by maintaining epidermal growth factor receptor (egfr) stability and egfr/p38 signalling activation. Acta Physiol..

